# Exploration of new models for primary dysmenorrhea treatment: low-power visible-light-activated photodynamic therapy and oral contraceptives

**DOI:** 10.3389/fmed.2024.1388045

**Published:** 2024-05-01

**Authors:** Yue Wang, Jie Chen, Zhiyuan Zhang, Xuesong Ding, Jingwen Gan, Yingying Guo, Wanqi Liang, Yanfang Wang, Yan Deng, Aijun Sun

**Affiliations:** National Clinical Research Center for Obstetric and Gynecologic Diseases Department of Obstetrics and Gynecology, Peking Union Medical College Hospital, Chinese Academy of Medical Sciences and Peking Union Medical College, Beijing, China

**Keywords:** primary dysmenorrhea, photodynamic therapy, oral contraceptive, Marvelon, quality of women's life

## Abstract

**Background:**

Primary dysmenorrhea (PD) is one of the most common reasons that affect the life quality of women during childbearing age. This research aims to explore the efficacy and curative effect characteristics of oral contraceptives and low-power visible-light-activated photodynamic therapy (PDT). Besides investigating the possible mechanism of PDT, we expected to find a treatment model with better efficacy and fewer side effects.

**Method:**

It was a multicenter, randomized, parallel-controlled study. Eligible participants were randomly assigned to three groups: placebo group, oral contraceptive (Marvelon) group, and the PDT group. They were treated continuously for three menstrual cycles and followed up for two cycles after treatment. The scores of the visual analog scale (VAS) and the concentration of pain-related small molecules in blood before and after treatment were recorded in each group, which can evaluate the therapeutic characteristics of different treatments.

**Result:**

Both Marvelon and PDT were effective. The effect of Marvelon appears quickly which can significantly relieve symptoms at the beginning, while PDT shows a relatively slow role. There was no significant difference in the final efficacy two cycles after treatment. The therapeutic effect was achieved by reducing the concentrations of prostaglandin 2 (PGE2) and endothelin (ET) in the blood.

**Conclusion:**

Marvelon and PDT are effective methods for the treatment of PD. The long-term efficacy of the two is similar, while the therapeutic characteristics and the side effects are different. Patients can choose the suitable way according to their individual needs.

## Graphical abstract

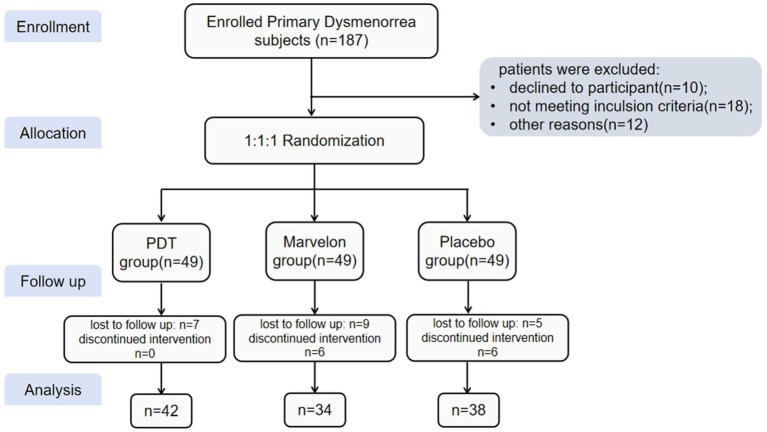

## Background

Dysmenorrhea is a major disease plaguing woman of reproductive age, mainly manifested in lower abdominal pain and swelling before, after or during menstruation, accompanied by lumbago or other discomfort, affecting 50–90% of women, which the most important cause of chronic pelvic pain (1). Dysmenorrhea can be divided into primary and secondary dysmenorrhea. Primary dysmenorrhea is a periodic pain associated with menstruation or ovulation. The etiology has not been fully defined, and the most widely accepted explanation is excessive production of uterine prostaglandins (2). The decrease of premenstrual progesterone causes endometrial shedding and contributes to inflammatory response. The prostaglandin released by cell decomposition is enhanced which stimulates muscle contraction, thus leading to uterine muscle ischemia and hypoxia, which eventually results in pain (3). Prostaglandin F2α (PGF2α) and prostaglandin E2 (PGE2) have particular roles in the inflammatory process. PGF2α mediates constriction of arcuate blood vessels, leading to local hypoxia of endometrial tissue. Another effect of PGF2α is to stimulate smooth muscle contraction, which in turn supports menstrual bleeding. The effect of PGE2 depends on the type of receptor, which includes relaxation of endometrial blood vessels and may increase swelling and recruitment of leukotrienes (4). Lundstrom (5) and Stromberg et al. (6), both found that women with dysmenorrhea had higher levels of prostaglandin in endometrium and plasma. However, the study of Liedman et al. (7) did not find any obvious anomaly. In addition, some small molecules such as vasopressin (VP) (6, 8), Tumor necrosis factor-α (TNF-α) (9), Interleukin-6 (IL-6) (10), vascular endothelial growth factor (VEGF) (11), and C-reactive protein (CRP) (12) may be associated with dysmenorrhea, but the evidence is inexact. Non-steroidal anti-inflammatory drugs (NSAIDs) are the first-line treatment for dysmenorrhea. NSAIDs work by inhibiting cyclooxygenase (COX), an enzyme responsible for prostaglandin synthesis (13). However, there is still a 20–25% failure rate, because some women are intolerant to it (13). A second possibility for treating dysmenorrhea is hormonal medicine, especially combined oral contraceptives, which work by limiting the growth of the endometrial lining, thus reducing the endometrial production of prostaglandins and leukotrienes. Oral contraceptives also work by inhibiting ovulation and thus progesterone production, which reduces the synthesis of prostaglandins and leukotrienes (14). Oral contraceptives with lower hormone doses were being used, which reduced the risk of adverse reactions. However, these doses may still increase the risk of breast cancer or venous thrombosis (15, 16).

At present, many researchers are exploring feasible physical therapy, whose biggest advantage is reducing the side effects brought by drugs (17). For example, Machado et al. (18) studied the influence of hyperthermia and transcutaneous electrical nerve stimulation on dysmenorrhea. Our previous studies (19) also confirmed that low-power visible-light-activated photodynamic therapy (PDT) can relieve primary dysmenorrhea to a certain extent, but there is no reliable controlled evaluation evidence of efficacy.

Based on current research status and existing treatment options, this study aims to confirm the small molecules associated with dysmenorrhea and compare the efficacy of photodynamic therapy with oral contraceptives, with a view to improving efficacy while reducing side effects.

## Method

### Study design and participants

The study was a multicenter, prospective, randomized, double-blind controlled trial to evaluate the efficacy and safety of PDT and Marvelon in the treatment of primary dysmenorrhea. The study protocol has been reviewed and approved by the institutional review committees of the eight participating hospitals (No. ZS-1913), which has been registered with ClinicalTrials.gov (NCT03953716). Participants were recruited from seven provinces in China between March 2019 and August 2020 and received written informed consent from each participant. This research is in line with the Declaration of Helsinki. Patients aged 18–35 years, with informed consent, diagnosed with primary dysmenorrhea, and with normal menstrual cycles (21–35 days) were eligible for this study. The exclusion criteria are as follows: a. patients with irregular menstruation, who have influence on treatment and efficacy judgment; b. Patients who have used related drugs in the past 3 months; c. Substance abuse or dependence (alcohol or drugs) within the previous 3 months or Heavy smokers; d. Serious or unstable physical diseases, including liver, kidney, gastrointestinal tract, cardiovascular, respiratory, endocrine, nervous, immune or blood system, neuropsychiatric system, etc.; e. Lactating or pregnant women, or women within 1 year after delivery; f. previous allergy to the test drug or photosensitivity; g. A history of thromboembolic disease or a tendency to thrombosis.

### Intervention

This was a multicenter, randomized, open, parallel-control study. Based on their randomly selected numbers, participants were randomly assigned to three groups in a ratio of 1:1:1, namely the Photodynamic therapy (PDT) group, the Oral contraceptives (Marvelon) group, and the placebo group. Then, different groups were treated accordingly to evaluate and compare the clinical efficacy and safety. The possible mechanism was explored by serological analysis.

**Figure 1:**
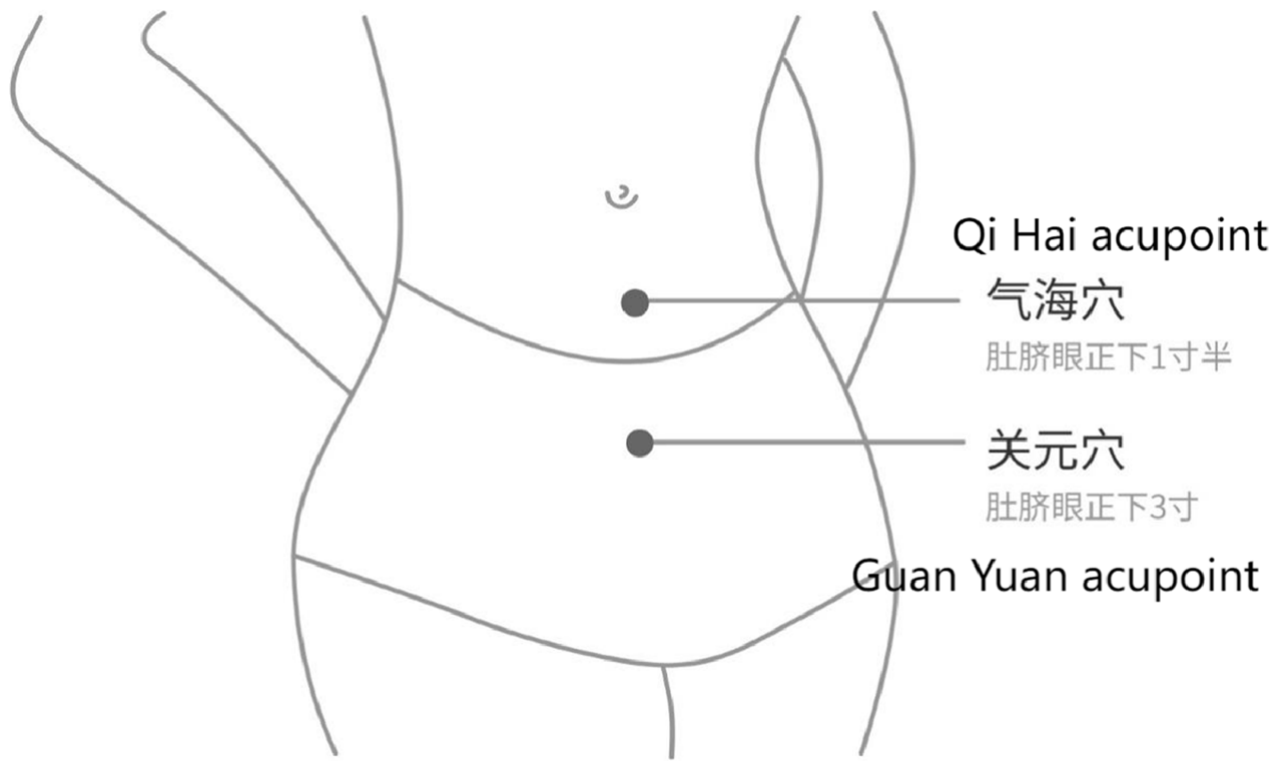
Acupoint distribution.

The use of PDT devices has been detailed in the previous article (19). Participants in the PDT group first need to download the Eospal Yuban mobile APP. Find the acupoints of “Qi Hai” and “Guan Yuan” (Figure 1). Apply a medical gel sheet to the above points. After connecting the host and light sensor, connect the light sensor to the patch. Turn on Bluetooth, match the mobile phone and PDT device, participants can control PDT through the mobile phone app, and the background can display the PDT treatment time. Since the end of menstruation, once a day, each time for 20 min, 5 days as a treatment cycle, each treatment cycle interval of 2 days, until the onset of menstruation. Use 3 menstrual cycles.

In the oral contraceptive group, Marvelon was taken orally. Each tablet contained 0.15 mg deoxygestrel and 30 μg ethinylestradiol. 1 tablet a day from the first day of the menstrual cycle for 21 consecutive days, followed by 7 days of withdrawal. Use 3 menstrual cycles.

The treatment regimen for the placebo group is provided here: Placebo 3.5gBID x 10d (for 10 days and starting 3–5 days before menstruation). The volunteers took the medication with warm water and used in 3 menstrual cycles.

A power analysis (two-sample t-test, performed in PASS software) showed that a sample size of 108 women would detect a difference in pain reduction of 3 points weighed by the visual analog scale (α = 0.05 and 1 − β = 0.90), a downgrade in pain (from severe to moderate or from moderate to mild). Thus, with a 15% of dropout rate, a total of 127 participants were needed in this trial (42 patients in each group). In the screening of actual patients, patients who did not follow up as required were excluded, and 114 participants were included in the final analysis.

### Evaluation

As the primary endpoint index, the pain intensity of PD was assessed on either day 1 or day 2 of menstruation using a visual analog scale (VAS) ranging from 0 (no pain) at baseline to 10 (most severe pain) every 4 weeks. Pain intensity was ranked according to the VAS score, which was 1–3 for mild pain, 4–6 for moderate pain, and 7–10 for severe pain. SF-MPQ, a simplified version of the MPQ Pain Scale, maintained 11 pain intensity assessments and 4 pain-emotion items, which enabled the detailed recording of the characteristics, intensity, emotional state, and psychological experience of pain. A score of 0–3 indicates “no, light, moderate, and severe” pain of different degrees. The pain rating index can be calculated from the sum of the scores. The Cox Menstrual Symptom Scale (CMSS) included local and systemic symptoms, and the duration of symptoms was evaluated comprehensively. All items use a 5-level scoring method. Severity: 0- no discomfort, 1- mild discomfort, 2- moderate discomfort, 3- severe discomfort, 4- very serious; Duration: 0- none, 1- lasts <3 h, 2- lasts 3 ~ 7 h, 3- lasts 7 ~ 24 h, 4- lasts >24 h. Symptoms were scored for severity and duration.

The secondary evaluation index included the level of PGE2, PGF2α and other small molecules in peripheral blood before and after treatment. All these molecules were quantified by enzyme-linked immunosorbent assay (ELISA) according to the manufacturer’s instructions (RENJIE Biotech Inc., Shanghai, China). Blood samples were collected at baseline menstrual cycle day 2 and week 12 of treatment. The samples were centrifuged at 3000 RPM for 20 min, then the supernatant was stored at −80°C. The serum thaws at room temperature.

Prostaglandins play an important role in primary dysmenorrhea and are considered to be an important mediator. In women with primary dysmenorrhea, prostaglandin levels are always higher. Prostaglandins may cause contractions of the uterus (as they do during childbirth), resulting in reduced blood flow to the uterus. This contraction can cause pain and discomfort. Prostaglandins also cause nerve endings in the uterus to sense pain more clearly. We performed imaging assessments of the pulsatile index and resistance index of blood flow in major uterine vessels before and after treatment to identify possible differences.

### Statistical analysis

Participants who completed the entire treatment and all study assessments were included in the statistical analysis. The data were expressed as mean ± standard error (SEM). Repeated measurements were used to analyze the VAS scores between the three groups. Independent sample *t*-test was used to compare the basic characteristics, changes in biochemical factors and uterine artery blood flow index among all groups. The paired T-test was used to determine the intra-group difference in the longitude comparison between the base value and the end result. Statistical analysis was performed using SPSS version 23.0 (IBM, Armonk, NY, USA). *p* < 0.05 was considered statistically significant.

## Results

Participant recruitment was conducted under the supervision of health professionals. Excluding the lost participants, 114 patients were included in the study, including the placebo group (*n* = 38), Marvelon group (*n* = 34), and PDT group (*n* = 42). Participants completed 12 weeks of study and post-treatment assessment and were included in the full analysis. At the start of the study, participants in the three groups matched well. The demographic characteristics are shown in Table 1. There were no significant differences in age, body mass index (BMI), menarche time, menstrual cycle, symptom duration and VAS score. The period duration of the placebo group was slightly shorter than that of the Marvelon and PDT groups, but all three groups were within the normal range.

**Table 1:** Demographic characteristics of participants.

Characteristics	Overall (*n* = 114)	Placebo (*n* = 38)	Marvelon (*n* = 34)	PDT (*n* = 42)	*p* value
Age, yr	26.04 ± 4.66	26.00 ± 4.61	25.56 ± 4.86	26.47 ± 4.61	0.697
BMI, kg/m^2^	20.47 ± 3.48	20.94 ± 4.31	19.93 ± 2.29	20.50 ± 3.46	0.478
Age at menarche, yr	13.08 ± 1.28	13.11 ± 1.29	13.15 ± 1.48	13.00 ± 1.10	0.874
Menstrual cycle, days	29.75 ± 2.65	29.50 ± 2.86	29.74 ± 2.49	29.98 ± 2.61	0.727
Symptom duration, days	12.96 ± 4.49	12.89 ± 4.47	12.41 ± 4.53	13.48 ± 4.52	0.590
Bleeding duration, days	5.99 ± 1.27	5.53 ± 1.37	6.21 ± 1.09	6.24 ± 1.23	0.021^a,b^
VAS scores	5.32 ± 2.70	5.45 ± 2.65	5.38 ± 2.74	5.17 ± 2.78	0.890

P^a^, Placebo group vs Marvelon group. P^b^, Placebo group vs PDT group.

### Symptom improvement

**Figure 2:**
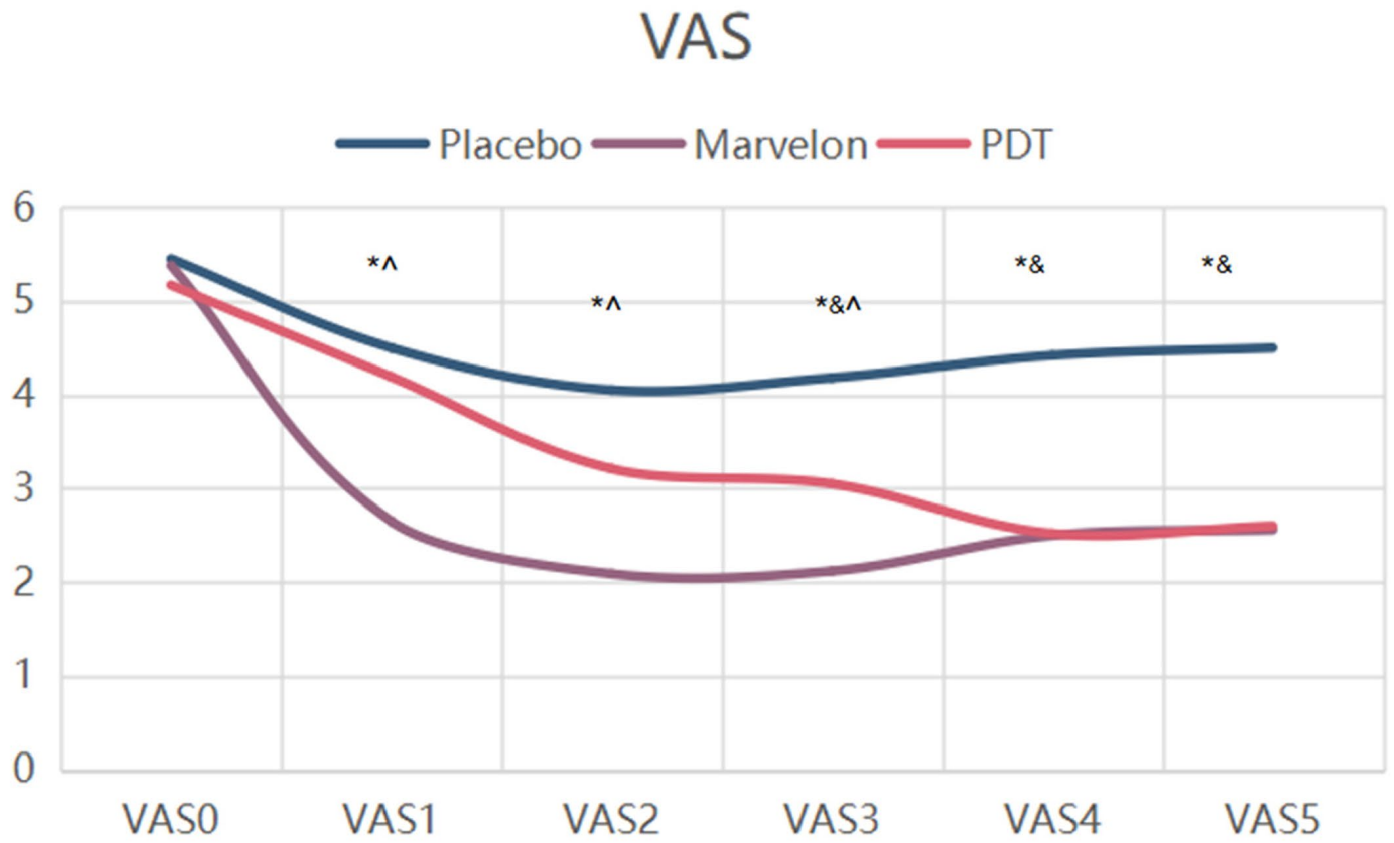
Changes in VAS scores during treatment. p*, Placebo group vs. PDT group; p^, PDT group vs. Marvelon group; p&, Marvelon group vs. Placebo group.

The absolute change in VAS scores for all participants is shown in Figure 2. The score was divided into 6 groups, namely VAS 0, VAS 1, VAS 2, VAS 3, VAS 4 and VAS 5. VAS 0 was before treatment, VAS 1, 2 and 3 were after 1, 2 and 3 therapeutic cycles, and VAS 4 and 5 were after 1 and 2 cycles of therapy, respectively. Compared with placebo, VAS scores were significantly lower in the Marvelon and PDT groups (*p* < 0.001). The effect of PDT group is characterized by slow development of curative effect. While the Marvelon group experienced significant relief of symptoms within the first cycle of treatment (*p* < 0.05). Although the efficacy of Marvelon was better than that of the PDT group at the end of the first cycle of treatment, the efficacy was similar at the end of three cycles (VAS 3) of treatment and two cycles after treatment (VAS 5).

**Figure 3:**
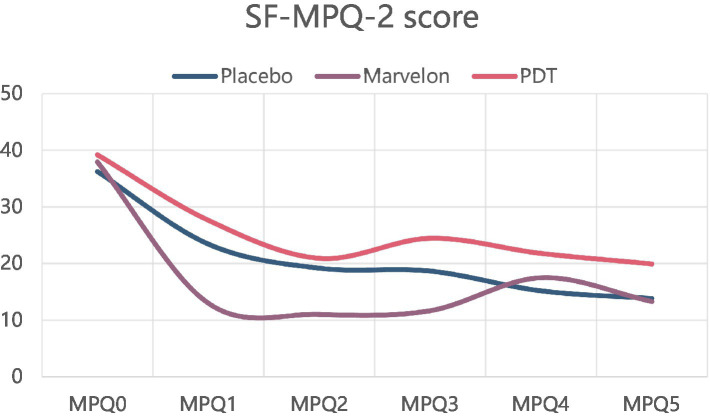
Changes in SF-MPQ-2 scale during treatment.

**Figure 4:**
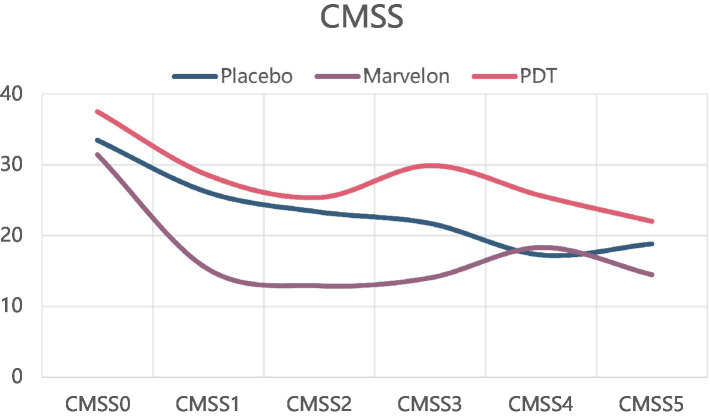
Changes in CMSS scale during treatment.

While on the simplified McGill Pain Questionnaire (SF-MPQ) and CMSS scores, the PDT group and Marvelon group showed no advantage. All three groups showed significant symptom relief (Figures 3, 4).

### Biochemical index

All participants underwent blood biochemical monitoring during the treatment (Table 2). In addition to PGE2 and PGF2α, which are associated with pain, small molecules such as endothelin (ET), vasopressin (VP), nitric oxide (NO), beta-endorphin (βEP) and oxytocin (OT) have been included in our research. Through the analysis of differences (d) before and after treatment, both PGE2 and ET in the Marvelon group and the PDT group were significantly decreased after treatment, and the disparities were statistically significant compared with the placebo group. However, our study did not detect changes in PGF 2α.

**Table 2:** Changes of serological indexes in each group before and after treatment.

Molecular	Placebo group			Marvelon group			PDT group			*p* value
	Before	After	Dif.	Before	After	Dif.	Before	After	Dif.	
PGE 2 (pg/mL)	590.66	520.03	70.63	558.35	452.81	105.54	592.21	482.50	109.71	0.020^a,b^
PGF 2α (pg/mL)	43.17	38.55	4.62	45.01	40.54	4.47	44.48	39.15	5.33	0.710
ET (pg/mL)	156.33	139.34	16.99	156.69	131.07	25.62	154.98	128.19	26.79	0.000^a,b^
VP (pg/mL)	1650.99	1497.75	153.24	1637.50	1434.82	202.68	1620.44	1425.83	194.61	0.439
NO (μmol/L)	7.62	7.58	0.04	7.84	7.82	0.02	8.35	7.26	1.09	0.254
βEP (pg/mL)	28.64	32.56	−3.86	29.42	34.27	−4.85	28.13	32.99	−4.86	0.364
OT (pg/mL)	209.54	188.29	21.25	226.22	200.89	25.33	216.10	188.02	28.08	0.420

P^a^, Placebo group vs Marvelon group. P^b^, Placebo group vs PDT group.

### Ultrasonic index

Gynecologic ultrasound, as an indicator of efficacy and safety, was monitored before and after treatment. The results are shown in Table 3. All the indicators of Doppler blood flow in the three groups decreased slightly before and after treatment, but there was no statistical significance. This also means that no physical change has taken place.

**Table 3:** Changes of ultrasound Doppler blood flow before and after treatment.

	Placebo group			Marvelon group			PDT group			*p* value
	Before	After	Dif.	Before	After	Dif.	Before	After	Dif.	
L-PI	2.51	2.24	0.27	2.40	2.09	0.31	2.61	2.16	0.45	0.542
R-PI	2.55	2.22	0.33	2.64	2.15	0.49	2.51	2.28	0.23	0.352
L-RI	0.84	0.82	0.02	0.85	0.80	0.05	0.84	0.81	0.03	0.508
R-RI	0.81	0.81	0	0.84	0.80	0.04	0.84	0.81	0.03	0.158
L-S/D	6.74	6.13	0.61	6.72	5.52	0.20	6.64	5.79	0.85	0.493
R-S/D	6.98	5.79	1.19	6.91	5.54	1.37	7.04	5.97	1.07	0.956

PI, left uterine artery Pulsation index; R-PI, right uterine artery Pulsation index; L-RI, left uterine artery Resistance index; R-RI, right uterine artery Resistance index; L-S/D, left uterine artery S/D; R-S/D, left uterine artery S/D.

## Discussion

Primary dysmenorrhea is a major category of diseases affecting the quality of women’s life of reproductive age. The cause of the disease is not clear, and there is a lack of effective radical treatment. So, the treatment method is still controversial. How to relieve primary dysmenorrhea safely and effectively is also an important direction of clinical research. In this study, a multicenter, randomized, open, parallel controlled design was used to find a treatment with reliable efficacy and fewer side effects and to explore the possible mechanism by monitoring serum small molecule indicators.

In terms of symptom indicators, the most significant result was the decrease in VAS score. VAS score was significantly decreased in both the Marvelon group and the PDT group, which could last for a period of time until the end of treatment. The difference between the two groups was that the Marvelon group showed a significant analgesic effect at the beginning of treatment, while the PDT group gradually showed a role in the treatment. However, there was no significant change in SF-MPQ-2 and CMSS scores. The VAS score is one of the most commonly used pain evaluation systems, focusing at the severity of the pain itself, while the other two score systems combine scores of emotional symptoms associated with pain, taking more time to complete. Therefore, they are likely to be subjected to more confounding factors. Two conclusions can be drawn from this. First of all, as far as the simple analgesic effect is concerned, the curative effect of Marvelon and PDT is considerable. Secondly, primary dysmenorrhea is a result of both organic and psychological factors. This was demonstrated by the placebo effect alone, which improved the severity of symptoms in most participants.

Changes in PGE2, PGF2α, ET, VP, NO, β-EP and OT were monitored in our study. At the time of enrollment, there was no significant difference in the level of small molecules among the three groups. The efficacy was evaluated by comparing the differences before and after treatment. With the relief of symptoms, PGE2 and ET both showed a significant decrease. The reduction in the PDT group and the Marvelon group was statistically significant compared with the placebo group. Prostaglandin E2 (PGE2) is a lipid signaling molecule involved in pain and inflammation, which may promote tissue regeneration and repair after injury of different organ systems (20). The main ingredients of Marvelon include desogonolone and ethinyl estradiol, and side effects have been found to include irregular menstruation, nausea, decreased appetite, and in a small number of patients, blood clots in the lower extremities after treatment. In clinical practice, non-steroidal anti-inflammatory drugs (NSAIDs) are most commonly used to reduce PGE2. However, some existing studies have shown that this method usually has side effects on the tissue repair process. Such as bone repair, kidney tissue repair, etc. (21–24), it also has a certain impact on fertility (25).

Endotoxin (ET) is a kind of lipopolysaccharide associated with inflammation. The study of Baker M and Wiedermann CJ et al. determined that the average level of ET in the normal population was 10 ± 20 pg./mL (26), and other studies have also confirmed that the level of ET was significantly increased in atherosclerosis, amyotrophic lateral sclerosis, cirrhosis, peritonitis and other diseases (27). Endotoxin causes inflammatory activation mainly by activating TLR4 (co-receptor MD2) on the cell surface, leading to transcription of NF-κB to activate hundreds of inflammatory genes, including pro-inflammatory cytokines such as TNF-α, IL-6 and pro-IL-1β (28). Intravenous injection of 1ngLPS/kg (equivalent to 15 pg./mL distributed through the blood) into healthy human volunteers has been shown to result in an increase in blood cytokines (TNFα, IL-6, IL-8, IL-10) within 1–3 h. Disease behavior (fatigue, headache, muscle pain, tremors) and motivation (alertness, energy, attention, vitality, social interest) were reduced and reversed within 4 h (29). PGF2α is also one of the molecules closely related to primary dysmenorrhea. However, in our study, PGF2α decreased in all groups after treatment, but there was no significant difference, which may be related to insufficient sample size and other factors.

Ultrasound is mainly used to monitor blood flow changes and provide further safety verification indicators for treatment. The uterine artery is the main blood supply to the uterus. Existing studies have shown that low-dose aspirin, heparin, progesterone and nitric oxide donors (such as isosorbide mononitrate) can effectively improve uterine arterial blood flow, thus affecting pregnancy outcome. Our study did not find any organic changes in the uterus before and after treatment, suggesting that no matter what kind of treatment is used, it is relatively safe in the short term.

To explore the mechanism of therapeutic instruments, Chinese researchers believe that acupoints are a complex of skin, muscle and nerve, with dense innervation. Photodynamic therapy is based on the possibility of selectively destroying pathological tissue by using accumulated photosensitizers (30), namely stimulating uterine tissue cells, soothing uterine smooth muscle, restoring blood circulation of uterine tissue and enhancing the metabolism of damaged uterine muscle cells, thus reducing pain. The onset of PDT was a little later than that of Marvelon, which partially confirmed the cumulative effect that was required during the course of the treatment. The “acupoint” selective treatment of traditional Chinese medicine combined with the local action of photodynamic, two factors jointly determine the local efficacy of the therapeutic instrument, and the side effects on other systems and tissues of the whole body are less than that of western medicine, which is also the main reason why we choose this treatment method.

## Conclusion

The research confirmed that both Marvelon and PDT treatment can relieve the symptoms of primary dysmenorrhea. The former works quickly, but there are potential side effects. The latter one is slower to take effect but has relatively few side effects. They work by inhibiting PGE2 and ET, and their effects persist two cycles after the end of treatment. Different patients can choose their own way according to the treatment characteristics of different methods. Our study also has corresponding limitations. The downstream molecules of the pathway where PGE2 and ET are located were not measured and tracked. Besides, we only speculated that TNFα, IL-6, pro-IL-1β and other molecules might be used for anti-inflammatory and pain relief. However, there may be other undiscovered pathways that need further research to explore.

## Data availability statement

The original contributions presented in the study are included in the article/supplementary material, further inquiries can be directed to the corresponding author.

## Ethics statement

This trial was approved by the Ethics Committee of Peking Union Medical College Hospital, Peking Union Medical College, Chinese Academy of Medical Science (No. ZS-1913). All participants provided written informed consent. The studies were conducted in accordance with the local legislation and institutional requirements. The participants provided their written informed consent to participate in this study.

## Author contributions

YuW: Conceptualization, Data curation, Investigation, Methodology, Writing – original draft. JC: Conceptualization, Writing – original draft. ZZ: Data curation, Formal analysis, Writing – original draft. XD: Investigation, Software, Writing – original draft. JG: Conceptualization, Investigation, Software, Writing – original draft. YG: Formal analysis, Funding acquisition, Project administration, Resources, Validation, Visualization, Writing – original draft. WL: Formal analysis, Project administration, Validation, Writing – original draft. YaW: Data curation, Methodology, Supervision, Writing – original draft. YD: Conceptualization, Investigation, Software, Writing – original draft. AS: Conceptualization, Data curation, Formal analysis, Funding acquisition, Investigation, Methodology, Project administration, Resources, Software, Supervision, Validation, Visualization, Writing – original draft, Writing – review & editing.
